# Supplementary material for: Identification of photoperiod-induced specific miRNAs in the adrenal glands of Sunite sheep (Ovis aries)
Source: Front Vet Sci. 2022 Jul 22;9:888207. doi: 10.3389/fvets.2022.888207 (PMC9354845; doi:10.3389/fvets.2022.888207)
Supplement: Supplementary file 1 [file Data_Sheet_1.ZIP › Supplementary file/miRDeep2 file.zip!/miRDeep2 file/Know_Structure/SPLP42A2/oar-mir-150.pdf]

miRBase precursor : oar-mir-150
Total read count : 772
oar-miR-150 read count : 770
remaining reads : 2

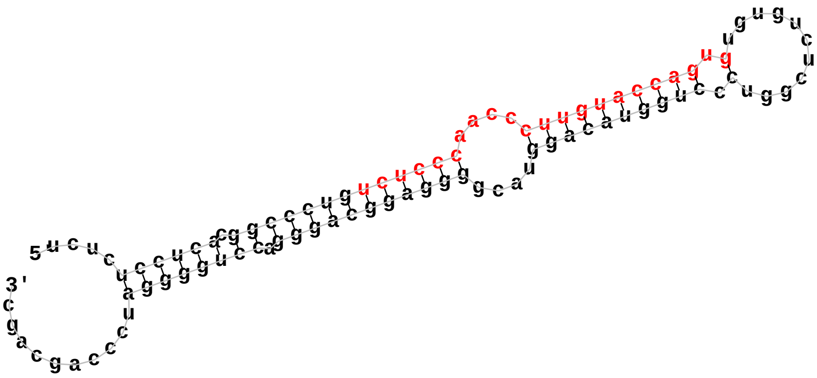

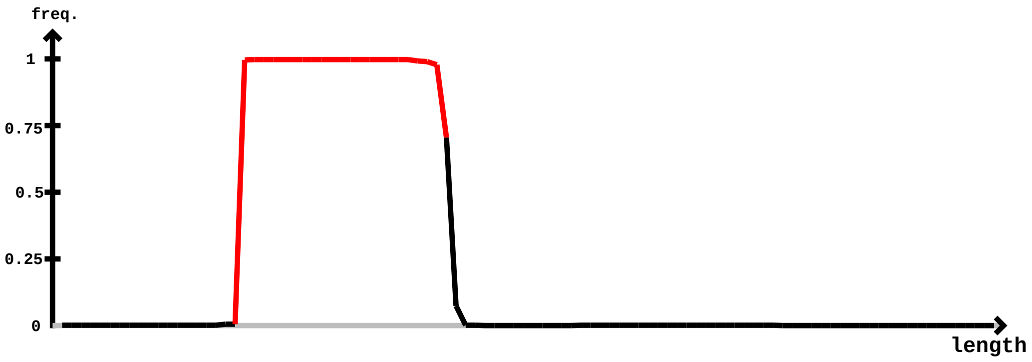

oar-miR-150

```
5'- ucucuccucacggcccugucucccaacccuuguaccagugugugucucggucccugguacagguacggggaggcagggaccuggggaucccagcagc   -3'   exp
    ....((((((.(((((((((((((....((((((((((.(...........).))))))))))....))))))))))).))))))))..........    reads   mm   sample
    ucucuccucacggcccug.............................................................................    1       0    seq
    ................ugucucccaacccuuguaccagu..........................................................    3       0    seq
    ..................ucucccaacccuuguacc.............................................................    4       0    seq
    ..................ucucccaacccuuguacca............................................................    2       0    seq
    ..................ucucccaacccuuguaccag...........................................................    9       0    seq
    ..................ucucccaacccuuguaccagu..........................................................    208     0    seq
    ..................ucucccaacccuuguaccagug.........................................................    486     0    seq
    ..................ucucccaacccuuguaccagugu........................................................    56      0    seq
    ..................ucucccaacccuuguaccagugugu......................................................    1       0    seq
    ...................cucccaacccuuguaccagug.........................................................    1       0    seq
    .....................................................cugguacagguacggggaggc.......................    1       0    seq
```
